# RETRACTED: Rangasamy et al. Protection of Mice from Controlled Cortical Impact Injury by Food Additive Glyceryl Tribenzoate. *Int. J. Mol. Sci.* 2023, *24*, 2083

**DOI:** 10.3390/ijms27073040

**Published:** 2026-03-27

**Authors:** Suresh B. Rangasamy, Jit Poddar, Kalipada Pahan

**Affiliations:** 1 Division of Research and Development, Jesse Brown Veterans Affairs Medical Center, Chicago, IL 60612, USA; 2 Department of Neurological Sciences, Rush University Medical Center, Chicago, IL 60612, USA; jit_poddar@rush.edu

The journal retracts the article “Protection of mice from controlled cortical impact injury by food additive glyceryl tribenzoate” [1] cited above.

Following publication, concerns were brought to the attention of the Editorial Office regarding the integrity of images presented in this publication [1].

In accordance with standard journal procedures, the Editorial Office and Editorial Board conducted an investigation that identified indications of inappropriate editing and partial duplication within Figure 3 presented in this article [1]. While the authors collaborated within this process, raw material meeting the journal’s requirements for original images could not be provided for evaluation by the Editorial Board. Consequently, the Editorial Board has lost confidence in the reliability of the findings and has decided to retract this publication, as per MDPI’s retraction policy.

This retraction was approved by the Editor-in-Chief of the journal *International Journal of Molecular Sciences*.

The authors have been informed of this retraction and have indicated their agreement with this decision.
